# Overcoming Breast Cancer Drug Resistance: A Novel Approach Using siRNA-Mediated P-glycoprotein Downregulation to Enhance Vinorelbine Efficacy

**DOI:** 10.34172/apb.2024.030

**Published:** 2024-03-09

**Authors:** Zahra Abbasfard, Abbas Behzad-Behbahani, Banafshe Rastegari, Sirous Naeimi, Mehdi Moghanibashi, Fatemeh Safari

**Affiliations:** ^1^Department of Genetics, College of Science, Islamic Azad University, Kazerun Branch, Kazerun, Iran.; ^2^Diagnostic Laboratory Sciences and Technology Research Center, School of Paramedical Sciences, Shiraz University of Medical Sciences, Shiraz, Iran.

**Keywords:** Drug resistance, Gene expression, MCF-7 cells, Multiple, Small interfering, Vinorelbine

## Abstract

**Purpose:**

Cancer, the second leading cause of mortality worldwide, represents a global health challenge, primarily due to drug resistance. Vinorelbine is a chemotherapeutic agent that disrupts cancer cell growth by targeting microtubules and inducing apoptosis. However, drug resistance remains a formidable obstacle. This resistance is caused by various factors including genetic mutations, drug efflux mechanisms, and DNA repair systems. Resolution of this challenge requires an innovative approach. This study investigated the potential of small interfering RNA (siRNA) to target and downregulate a vinorelbine-resistant MCF-7/ADR breast cancer cell line.

**Methods:**

Cells were cultured in Dulbecco’s modified Eagle’s medium (DMEM) 10% fetal bovine serum/penicillin/streptomycin. An siRNA targeting ABCB1 was designed and synthesized, and the cells were transfected with siRNA at final concentrations of 10, 20, and 30 nM. The3-(4,5-dimethylthiazol-2-yl)-2,5-diphenyltetrazolium bromide (MTT) assay was used to assess cell viability. ABCB1 mRNA expression levels were determined by real-time polymerase chain reaction (PCR).

**Results:**

MCF-7 cells exhibited a higher sensitivity to vinorelbine than MCF-7/ADR cells. MCF-7/ADR cells exhibited resistance to vinorelbine at concentrations, 12.50 and 25.00 μM. Treatment with siRNA significantly reduced *ABCB1* expression by 2.93-fold (*P*=0.0001). Similarly, co-treatment with siRNA and vinorelbine produced a substantial 2.89-fold decrease in ABCB1 gene expression in MCF-7 cells compared to that in MCF-7/ADR cells (*P*=0.0001).

**Conclusion:**

The results of the present study indicate that the concurrent use of siRNA and vinorelbine holds substantial promise as a therapeutic approach to overcome ABCB1-mediated multidrug resistance (MDR) in breast cancer. It is necessary to conduct comprehensive clinical trials to determine the true effectiveness of this combination therapy.

## Introduction

 Cancer is a major global contributor to mortality. Heart disease ranks as the second leading cause of death worldwide.^1^ One of the primary obstacles in cancer treatment is the emergence of drug resistance.^2^

 Vinorelbine is a chemotherapeutic medication used in the management of a range of cancers, including lung and breast cancers.^3,4^ Vino disrupts cancer cell growth by interfering with microtubules, which are crucial for cell shape and division.^5,6^ It inhibits mitosis by disrupting the microtubules and preventing chromosome separation. This leads to cell cycle arrest in the G2/M phase, thereby impeding cancer cell division.^7^ Vinorelbine can also induce apoptosis, programmed cell death, and elimination of damaged cells.^8^ This mechanism ultimately contributes to effective cancer treatment.

 Vinorelbine can become ineffective over time due to drug resistance. This is because cancer cells can develop mutations that render them less susceptible to drugs.

 Drug resistance is the ability of cancer cells to survive and grow despite exposure to chemotherapy drugs.^9-11^ This can happen for several reasons, including mutations in cancer cells that make them less susceptible to drugs,^12,13^ cancer cells developing ways to pump the drugs out of their cells,^14,15^ and cancer cells developing ways to repair the damage caused by the drugs.^16,17^ Drug resistance development can be influenced by additional factors such as the specific cancer type, stage, and overall health of the patient.

 The emergence of drug resistance is a significant obstacle in cancer treatment. Several approaches have been proposed to address this challenge. One approach is combination chemotherapy, in which multiple drugs are simultaneously employed. This strategy prevents cancer cells from acquiring resistance to drugs. Another approach is targeted therapy, which focuses on specific molecules essential for the growth and survival of cancer cell.^18^ Furthermore, immunotherapy connects the natural immune system to fight cancer. These methods have great potential for overcoming drug resistance in cancer treatments.^19,20^

 Small interfering RNA (siRNA) is a short strand of RNA that can be used to silence the expression of specific genes by binding and degrading them. This prevents the gene from being translated into a protein that silences its expression. In cancer, siRNA can be used to target genes involved in cancer cell resistance to chemotherapy.^21^ This can be achieved by designing siRNA that is complementary to the mRNA of genes involved in drug resistance.^22^

 siRNA is effective in reducing the growth of cancer cells, making them more sensitive to chemotherapy in animal studies.^8,23^

 Drug resistance in cancer cells is a multifaceted phenomenon and multiple genes play essential roles in this process.ATP-binding cassette (ABC) transporters are involved in several critical physiological and pathological phenomena like cell apoptosis, energy metabolism, detoxification, drug resistance, lymph-node metastasis, and poor survival in patients harboring breast tumors.^24-26^ *ABCDB1*, which belongs to the ABC transporter family, has recently garnered substantial attention because of its association with drug-resistance mechanisms. On the other hands, overexpression of *ABCB1 *was significantly related with the *IL6, CSF1, CSF3*, elevation. These factors, specially IL-6/JAK/STAT-3 pathway, cause drug resistance by inducing several pro-survival factors like surviving.^26^ Also, some classes of chemotherapy regiment like aflibercept (anti-VEGF) triggered autocrine secretion of IL-6 so that the resistance becomes more effective through of STAT-3 signaling hyperactivation.^26,27^ Previous works have shown that the metronomic administration of vinorelbine resulted in transient regression of T_reg_ cells which is mainly contributed in immune response suppression.^28^

 ABCDB encodes a protein called P-glycoprotein (P-gp), a membrane transporter that pumps chemotherapeutic agents out of cancer cells. This renders cancer cells resistant to chemotherapy because they cannot reach their targets. Overexpression or hyperactivity of *ABCDB1* is a significant problem in successful cancer treatment.

 MCF-7/ADR, formerly known as NCI/ADR-RES, is a multidrug-resistant breast cancer cell line that was derived from the MCF-7 cell line through selective breeding for resistance to the chemotherapy drug adriamycin (doxorubicin). MCF-7/ADR cells have proven to be an invaluable tool in breast cancer research, providing valuable insights into drug resistance mechanisms and contributing to the development of novel anti-cancer therapies.

 The main goal of this study was to explore the potential of siRNA as a targeted approach to downregulate ABCDB1 expression. This approach was intended to increase the sensitivity of vinorelbine-resistant cancer cell lines to treatment.

## Methods

###  Cell lines and cell culture

 The MCF-7 human breast cancer cell line and its ADR-resistant counterpart, MCF-7/ADR, were procured from the Cell Bank of the Tehran Pasture Institute in Tehran, Iran, and Lage’s laboratory at the François Jacob Biology Institute in Evry, France. The cell lines were obtained from ATCC (USA).

 Cell lines were maintained in Dulbecco’s modified Eagle’s medium (DMEM) (Thermo Fisher Scientific, USA) supplemented with 10% fetal bovine serum (FBS), 100 units/mL penicillin, and 100 μg/mL streptomycin (Gibco, USA). Incubation was incubated at 37°C in a humidified environment with 95% air and 5% CO2.

###  Transfection with siRNA

*ABCB1* siRNA was designed to target a specific nucleotide sequence in the ABCB1 coding region. FITC-labeled siRNAs were obtained from Bioneer Corporation (Daejeon, Korea). The sense sequence of each siRNA was 5′-CAGCAAAUAAGAACUGUGAUtt-3′ and the antisense sequence was 5′-AUCACAGUUCUAAUUGCUG-3′.

 MCF-7 and MCF-7/ADR cell lines were initially plated in 96-well plates, with each well containing 1 × 10^4^ cells, and in 6-well plates, with each well containing 2 × 10^5^ cells. These cells were incubated for 24 hours. On day 2, the cells were transfected with siRNA at final concentrations of 10, 20, and 30 nM using PEI as the transfection reagent (7 μL of 1 mg/mL transfection medium). siRNA and PEI were separately diluted in free DMEM and incubated at room temperature for 5 min. The prepared solutions were gently mixed and incubated for 45 min at room temperature. The mixtures were then added to the seeded cells. The cells were incubated for 24 hours at 37 °C in a humidified incubator containing 5% CO2. Following this incubation, the transfection medium was replaced with a complete medium.

###  Determining siRNA transfection efficiency

 We evaluated the cellular uptake efficiency of the FITC-labeled siRNA delivered by PEI using flow cytometry. In the initial step, we seeded both cell lines at a concentration of 4 × 10^4^ cells/well in a 24-well plate. FITC-labeled siRNA/PEI complexes were added to each well and incubated for 3 hours. Following incubation, the culture media containing transfection reagents was replaced with fresh complete media. The cells were washed with phosphate-buffered saline (PBS) and harvested by trypsinization. After two PBS washes, the cells were analyzed using a BD FACSCalibur flow cytometer. We determined the cellular transfection efficiency as the percentage of transfected cells compared to that of non-transfected cells, with the mean fluorescence intensity (MFI) serving as an indicator of FITC-labeled siRNA abundance within transfected cells.

###  Vinorelbine treatment and assessment of cell viability

In this study, we subjected MCF-7 and MCF-7/ADR cell lines to vinorelbine treatment, employing customized dosages for each cell line. to assess cell viability, we used the MTT assay. Initially, both cell lines were seeded at a concentration of 1 × 10^4^ cells/well in a 96-well plate and incubated overnight. Transfection was performed according to established protocols. Twenty-four hours after transfection, the cells were treated with vinorelbine (Sigma-Aldrich).

 The incubation period was extended to 24, 48, and 72 hours post-transfection, after which 0.5 mg/mL of 3-(4, 5-Dimethylthiazol-2-yl)-2,5-diphenyltetrazolium bromide (MTT) was administered for 4-hour duration. Formazan crystals were dissolved in dimethyl sulfoxide (DMSO), and the optical density (OD) was measured at 570 nm using a Stat-Fax 2100 Multimode Microplate Reader. To determine cell viability, we normalized the data obtained from treated cells to untreated cells using the MTT assay.

### Quantifying mRNA expression levels through real-time polymerase chain reaction (PCR)

 Our objective was to evaluate ABCBI mRNA expression levels in MCF-7 and MCF-7 ADR cells after treatment with the different regimens. Specifically, the cells were treated with *ABCB1* siRNA (20 nM) and vinorelbine (3.25 µM). After 48 hours of treatment, total RNA was extracted from cells using TRIzol reagent (Gibco, Life Technologies). cDNA was synthesized by reverse transcription (RT) using a PrimeScript RT Reagent Kit with random primers (TaKaRa).

 Quantitative real-time PCR was performed using primers specific for β-actin and ABCB1 (Origen Company). The PCR protocol used in this study consisted of an initial denaturation step at 95°C for 5 minutes, followed by 35 cycles of denaturation at 95 °C for 30 seconds, annealing at 62 °C for 30 seconds, and extension at 72 °C for 30 seconds. Comparative mRNA levels were estimated using the delta– delta CT (ΔΔCT) method. Table 1 lists the primer sequences of ABCB1 and β-actin genes.

**Table 1:** Primer sequences and amplified fragment lengths of ABCB1 and β-actin genes for real-time PCR assay

**Gene**	**Sequence**	**Amplified fragment length (bp)**
ABCB1	Forward: 5′- GCTGTCAAGGAAGCCAATGCCT-3′	120
	Reverse: 5′- TGCAATGGTCGTCGATCTCT-3	
β-Actin	Forward: 5′-ATCGTGCGTGACATTAAGGAG-3′	177
	Reverse: 5′-GAAGGAAGGCTGGAAGAGTG-3′	

###  Statistical analysis

 Statistical analyses were performed using GraphPad Prism version 8 (GraphPad Software, San Diego, California, USA). To investigate cellular toxicity, we conducted three separate repetitions of the MTT assay and analyzed the results using the mean ± standard deviation (SD) analysis. To assess the significance of variations in cell viability, and IC50 values within each group, we employed two-way ANOVA and non-parametric One-way ANOVA, respectively.

## Results

###  siRNA transfection efficiency 

**Figure 1:**
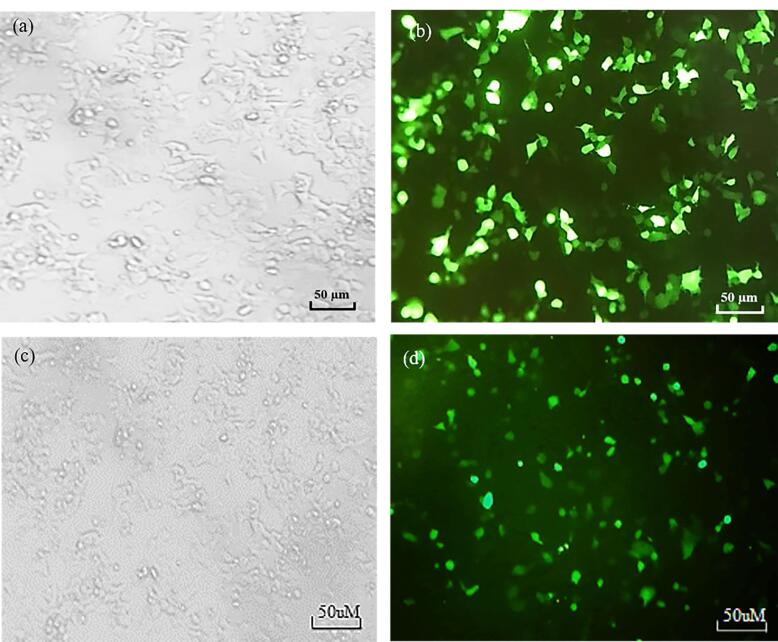
The siRNA transfection efficacy of MCF-7 cell line. a) The Bright Field and b) Green fluorescence microscopy images of MCF-7 cell line. c) The Bright Field and d) Green fluorescence microscopy images of MCF-7/ADR cell line

**Figure 2:**
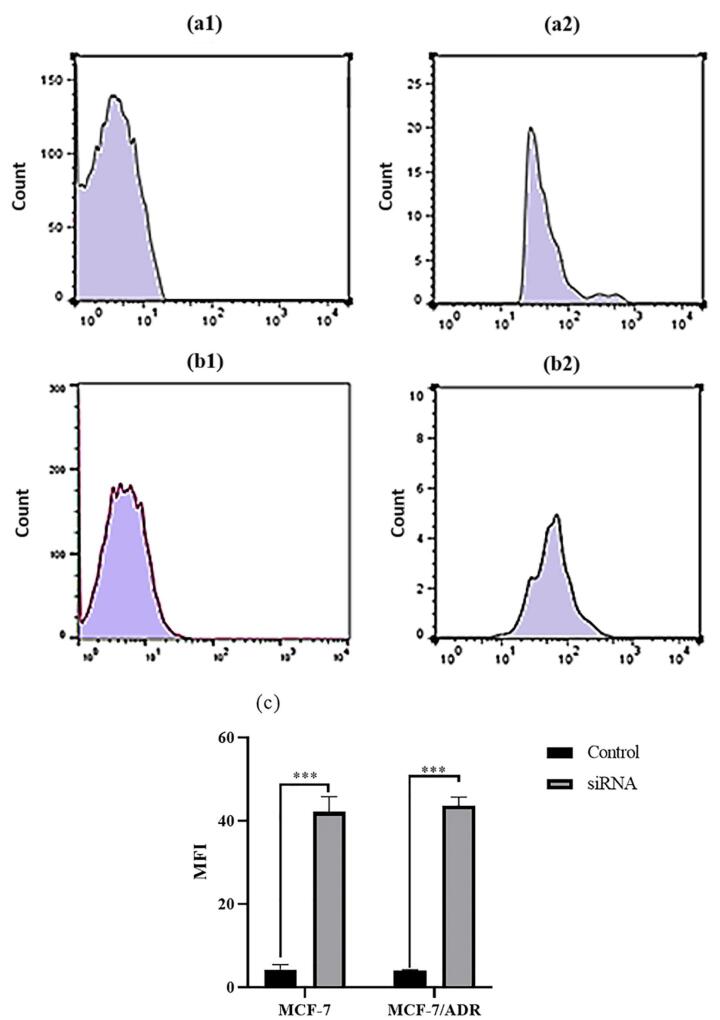
The fluorescence Intensity of siRNA transfection in MCF-7 and MCF-7/ADR cells. a1 and a2) The green fluorescence intensity of non-transfected and FITC-labeled siRNA-transfected of MCF-7 cells. b1 and b2) The green fluorescence intensity of non-transfected and FITC-labeled siRNA-transfected of MCF-7/ADR cells. C)Table of mean fluorescence intensities (MFIs) of non-transfected and transfected MCF-7 and MCF-7/ADR cells

 Transfection efficiency of FITC-tagged siRNA against *ABCB1* was assessed in MCF-7 and MCF-7/ADR cells. As illustrated in Figure 1, an siRNA concentration of 20 nM was selected as the optimum efficiency of transfection with approximately 70 % through fluorescence microscopy analysis for both cell lines. The green fluorescence intensity of the FITC-labeled siRNA was also measured in both MCF-7 and MCF-7/ADR cell lines using flow cytometry. The results showed high intensity of green fluorescence derived from siRNA transfection in both breast cancer cell lines (Figure 2).

###  Cell viability assay

 The MTT assay was employed to investigate the effects of vinorelbine on the MCF-7 and MCF-7/ADR cell lines.

**Figure 3:**
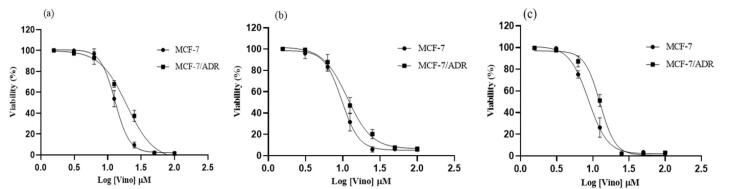
The fitting curve of the Vinorelbine treatment against MCF-7 and MCF-7/ADR cells after a) 24, b) 48, and c) 72 hours

 The cellular toxicity of vinorelbine was initially examined in MCF-7 and MCF-7/ADR cell lines at concentrations range from 1.57 to 100 μM, and the fitting curve results are presented in Figure 3. Vinorelbine treatment of MCF-7 and MCF-7/ADR cells resulted in severe toxicity at concentrations ≥ 50 μM. Also, 24-hour MCF-7 and MCF-7/ADR cell treatment at concentrations of 12.5 and 0.25 μM indicates the significant cellular resistance in the MCF-7/ADR cell at concentrations of 12.5 and 25.0 μM compared to the MCF-7 cell with a significant level of *P*= 0.02 and *P* < 0.0001, respectively (Figure 3a).

 Vinorelbine toxicity in MCF-7 and MCF-7/ADR cells within 48 hours also indicated severe toxicity at concentrations above 50 μM. MCF-7 and MCF-7/ADR cell treatment for 48 hours at 12.5 and 0.25 μM indicates a significant drug resistance in the MCF-7/ADR cell with the significant cellular resistance in the MCF-7/ADR cell at concentrations of 12.5 and 25.0 μM compared to the MCF-7 cell with a significant level of *P* = 0.011 and *P* = 0.025, respectively (Figure 3b).

 After 72 hours of treatment of MCF-7 and MCF-7/ADR cells with vinorelbine, toxicity was observed at concentrations ≥ 25 μM.

 At concentrations of 6.25 and 12.50 μM, a significant drug resistance in MCF-7/ADR cells was observed at significant levels of *P* = 0.0008 and *P* < 0.0001 compared with MCF-7 cells.

 IC50 value results for vinorelbine treatment are presented in Table 2. According to the results, the 48-h timeframe treatment was selected for further analysis.

**Table 2:** The IC50 values of vinorelbine against MCF-7 and MCF-7/ADR cancer cell lines

**IC50 (µM)**	**Time (h)**	**MCF-7**	**MCF-7/ADR**
Vinorelbine	24	12.61 ± 2.06	15.15 ± 1.24
	48	8.74 ± 1.61	11.31 ± 1.45
	72	8.50 ± 1.93	12.23 ± 1.53

The * and ** symbols represented the *P* values of 0.043, and 0.039, respectively.

###  Vinorelbine/siRNA combination therapy

**Figure 4:**
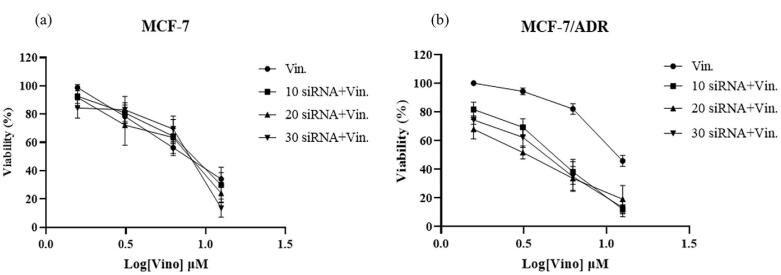
Cytotoxicity assessment of combination treatment of Vinorelbine and siRNA against MCF-7 (a) and MCF-7/ADR Cells (b)

 Combination therapy with vinflunine/siRNA has also been investigated. As shown in Figure 4a, no significant drug resistance was observed when 10, 20, and 30 nM of siRNA were used. However, in MCF-7/ADR cells, all siRNA concentrations significantly reduced drug resistance to various levels of vinorelbine. Based on these results, further investigations were conducted using 20 nM siRNA (Figure 4).

 In MCF-7/ADR cells, all siRNA concentrations reduced drug resistance at various levels of vinorelbine. Based on these results, further investigations were conducted using 20 nM siRNA.

 The IC50 values of the combination treatment of MCF-7 and MCF-7/ADR cells with different siRNA concentrations are presented in Table 3.

**Table 3:** IC50 values of Vinorelbine/siRNA treatment against MCF-7 and MCF-7/ADR cells

**siRNA (nM)**	**Time (h)**	**MCF-7**	**MCF-7/ADR**
-	48	8.93 ± 1.64	11.83 ± 1.38
10		7.97 ± 1.83	5.31 ± 1.49
20		8.11 ± 1.08	3.91 ± 0.53
30		7.94 ± 1.25	4.73 ± 1.90

The * and ** symbols represented the *P* values of 0.008, and 0.031, respectively.

###  Real-time PCR analysis 

**Figure 5:**
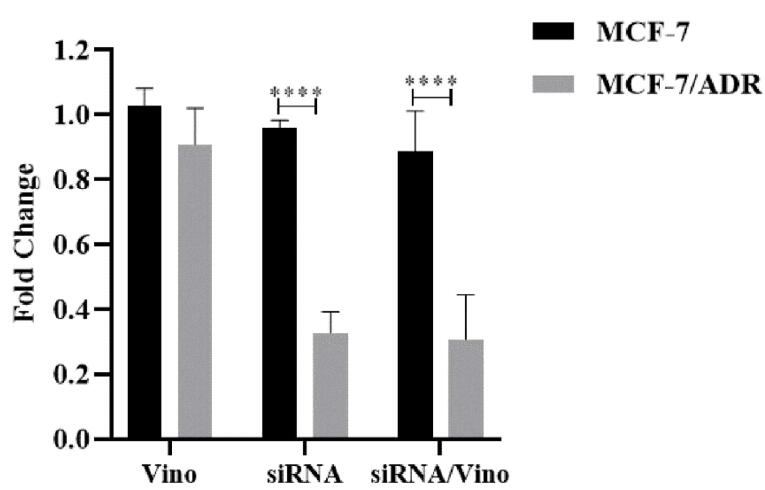
The fold change comparison of ABCB1 gene expression levels between MCF-7 and MCF-7/ADR cells after treatment with Vinorelbine, and simultaneous treatment of Vinorelbine with and without siRNA. The statistical analysis was performed using the One-Way ANOVA method, where significance levels were denoted as follows: * *P* ≤ 0.05, ** *P* ≤ 0.01, *** *P* ≤ 0.001, and **** *P* ≤ 0.0001

 SYBR Green-based Real-time PCR was used to investigate the impact of vinorelbine, siRNA, and their combinations on the expression of the *ABCB1* gene in MCF-7 and MCF-7/ADR cells. The results presented in Figure 5 demonstrate the following: When treated with vinorelbine, the MCF-7/ADR cell line showed minimal changes in *ABCB1* gene expression (0.97- and 1.02-fold, respectively) compared to the reference MCF-7 cell line. These changes were not statistically significant (*P* > 0.999 and *P* = 0.9139, respectively). In contrast, treatment with siRNA resulted in a significant reduction in *ABCB1* expression (*P* < 0.0001), with a change of 2.93-fold. Similarly, the combined treatment with siRNA and vinorelbine led to a significant decrease in *ABCB1* gene expression in the MCF-7 cell line relative to that in the MCF-7/ADR cell line, with a decrease of 2.89-fold (*P* < 0.0001). This intricate interplay ultimately underscores the potency of the treatment regimen in restricting ABCB1 expression in the MCF-7/ADR cell line.

## Discussion

 Vinorelbine is a chemotherapeutic drug used to treat a range of cancers, such as breast cancer, non-small cell lung cancer, ovarian cancer, and gastric cancer.^29^ Vinorelbine interferes with the growth and division of cancer cells. This occurs by binding to microtubules, which are structures that play a role in cell division. This binding prevents the formation of microtubules, which ultimately leads to cancer cell death.^30^ Cancer cells can become resistant to vinorelbine in several ways. One is through a process called multidrug resistance (MDR). This is when cancer cells develop proteins that pump vinorelbine out of the cells before they can bind to the microtubules.

 The successful development of effective therapeutic strategies against multidrug-resistant cancers, such as those mediated by overexpression of ABCB1, remains a critical challenge in the field of cancer treatment. In this study, we explored the potential of siRNA-based therapies in combination with the chemotherapeutic agent vinorelbine to combat drug resistance in breast cancer cells, particularly MCF-7/ADR cells with high *ABCB1* expression levels.

 Our results demonstrated the efficient transfection of FITC-tagged siRNA against the ABCB1 gene in both MCF-7 and MCF-7/ADR cell lines. This was evidenced by the approximately 70% transfection efficiency observed through fluorescence microscopy analysis at an siRNA concentration of 20 nM. Flow cytometry analysis further corroborated these findings, revealing a high green fluorescence intensity following siRNA transfection in both cell lines. These results indicate the successful delivery and uptake of siRNA, setting the stage for subsequent investigations of its therapeutic potential.

 Assessment of the cytotoxicity of vinorelbine on MCF-7 and MCF-7/ADR cells revealed noteworthy insights into drug resistance. At concentrations above 50 μM, severe toxicity was observed in both the cell lines, highlighting the potency of vinorelbine against cancer cells. However, a key observation emerged from our study - MCF-7/ADR cells displayed significant resistance to Vinorelbine treatment at concentrations of 12.5 and 25.0 μM, as compared to MCF-7 cells. This resistance was statistically significant, emphasizing the challenge of overcoming drug resistance in multidrug-resistant cancer cells. Limited studies have shown that the co administration of low-dose metronomic Vinorelbine with alpelisib regiment resulted in enhanced anti-cancer activity in MCF-7 cell lines.^31^

 An investigation into combination therapy with vinorelbine and siRNA revealed promising results.^32^ While no significant reduction in drug resistance was observed with siRNA concentrations of 10, 20, and 30 nM in MCF-7 cells, MCF-7/ADR cells exhibited a substantial decrease in resistance across various vinorelbine concentrations. Further analyses were conducted using 20 nM siRNA due to its effectiveness in overcoming drug resistance. The IC50 values of the combination treatment illustrated the potential of siRNA to sensitize MCF-7/ADR cells to vinorelbine, thereby enhancing cytotoxicity. Other reports have shown that siPgp and doxorubicin co-treatment; inhibited tumor growth and elevated drug uptake on MCF-7/MDR cells both in vitro and in vivo.^33^

 Evaluation of *ABCB1* gene expression through real-time PCR provided critical insights into the molecular mechanisms underlying our findings. Treatment with Vinorelbine alone resulted in minimal changes in ABCB1 gene expression in MCF-7/ADR cells compared to that in MCF-7 cells, which was statistically insignificant. In contrast, siRNA treatment significantly reduced ABCB1 expression, highlighting its potential to downregulate drug resistance-related genes. Remarkably, the combination of siRNA and vinorelbine synergistically decreased ABCB1 gene expression in MCF-7 cells compared to that in MCF-7/ADR cells, emphasizing the therapeutic advantage of this combinatorial approach.

 In conclusion, our study underscores the potential of siRNA-based therapies, particularly in combination with vinorelbine, in overcoming drug resistance in breast cancer cells with elevated ABCB1 expression. These findings suggest a promising strategy for improving the efficacy of chemotherapy for multidrug-resistant cancers. Further investigations into the mechanistic aspects of this combination therapy and its translation into clinical applications are warranted to elucidate its therapeutic potential.

## Conclusion

 In conclusion, the study demonstrates the potential of combining small interfering RNA (siRNA) with vinorelbine to overcome multidrug resistance (MDR) in breast cancer. The results show that siRNA targeting ABCB1 significantly reduces ABCB1 expression, thereby enhancing the sensitivity of MCF-7/ADR cells to vinorelbine. This combination therapy holds promise as a therapeutic approach to combat ABCB1-mediated MDR in breast cancer. Further comprehensive clinical trials are necessary to determine the true effectiveness of this combination therapy and its potential to improve treatment outcomes for patients with breast cancer.

## Acknowledgments

 The authors express their sincere gratitude to all members and staff of the Diagnostic Laboratory Sciences and Technology Research Center, School of Paramedical Sciences, Shiraz University of Medical Sciences, Shiraz, Iran, for their excellent help and cooperation in improving this study. We are also grateful to Miss Elina Mehdizadeh Naderi for native English editing of the manuscript. Specifically, we thank Gholamreza Rafiei Dehbidi and Farahnaz Zare for their valuable advice and suggestions.

## Competing Interests

 No potential conflict of interest was reported by the authors.

## Ethical Approval

 This study was approved by the Islamic Azad University-Kazeron Branch Ethics Committee (Ethics IR.IAU.KAU.REC.1402.074).

## References

[1] Siegel RL, Miller KD, Fuchs HE, Jemal A (2022). Cancer statistics, 2022. CA Cancer J Clin.

[2] Holohan C, Van Schaeybroeck S, Longley DB, Johnston PG (2013). Cancer drug resistance: an evolving paradigm. Nat Rev Cancer.

[3] Ghosh S, Javia A, Shetty S, Bardoliwala D, Maiti K, Banerjee S (2021). Triple negative breast cancer and non-small cell lung cancer: clinical challenges and nano-formulation approaches. J Control Release.

[4] Dhyani P, Quispe C, Sharma E, Bahukhandi A, Sati P, Attri DC (2022). Anticancer potential of alkaloids: a key emphasis to colchicine, vinblastine, vincristine, vindesine, vinorelbine and vincamine. Cancer Cell Int.

[5] Al-Mahayri ZN, Patrinos GP, Ali BR (2020). Toxicity and pharmacogenomic biomarkers in breast cancer chemotherapy. Front Pharmacol.

[6] Altinoz MA, Ozpinar A, Emekli-Alturfan E, Elmaci I (2018). Vinorelbine’s anti-tumor actions may depend on the mitotic apoptosis, autophagy and inflammation: hypotheses with implications for chemo-immunotherapy of advanced cancers and pediatric gliomas. J Chemother.

[7] Wordeman L, Vicente JJ (2021). Microtubule targeting agents in disease: classic drugs, novel roles. Cancers (Basel).

[8] Dhyani P, Quispe C, Sharma E, Bahukhandi A, Sati P, Attri DC (2022). Anticancer potential of alkaloids: a key emphasis to colchicine, vinblastine, vincristine, vindesine, vinorelbine and vincamine. Cancer Cell Int.

[9] Senga SS, Grose RP (2021). Hallmarks of cancer-the new testament. Open Biol.

[10] Aldea M, Andre F, Marabelle A, Dogan S, Barlesi F, Soria JC (2021). Overcoming resistance to tumor-targeted and immune-targeted therapies. Cancer Discov.

[11] Shi K, Wang G, Pei J, Zhang J, Wang J, Ouyang L (2022). Emerging strategies to overcome resistance to third-generation EGFR inhibitors. J Hematol Oncol.

[12] Mansoori B, Mohammadi A, Davudian S, Shirjang S, Baradaran B (2017). The different mechanisms of cancer drug resistance: a brief review. Adv Pharm Bull.

[13] Wang X, Zhang H, Chen X (2019). Drug resistance and combating drug resistance in cancer. Cancer Drug Resist.

[14] Muley H, Fadó R, Rodríguez-Rodríguez R, Casals N (2020). Drug uptake-based chemoresistance in breast cancer treatment. BiochemPharmacol.

[15] Borst P (2012). Cancer drug pan-resistance: pumps, cancer stem cells, quiescence, epithelial to mesenchymal transition, blocked cell death pathways, persisters or what?. Open Biol.

[16] Bukowski K, Kciuk M, Kontek R (2020). Mechanisms of multidrug resistance in cancer chemotherapy. Int J Mol Sci.

[17] Huang R, Zhou PK (2021). DNA damage repair: historical perspectives, mechanistic pathways and clinical translation for targeted cancer therapy. Signal Transduct Target Ther.

[18] Lee YT, Tan YJ, Oon CE (2018). Molecular targeted therapy: treating cancer with specificity. Eur J Pharmacol.

[19] Jia H, Truica CI, Wang B, Wang Y, Ren X, Harvey HA (2017). Immunotherapy for triple-negative breast cancer: existing challenges and exciting prospects. Drug Resist Updat.

[20] Ding Y, Wang Y, Hu Q (2022). Recent advances in overcoming barriers to cell-based delivery systems for cancer immunotherapy. Exploration (Beijing, China).

[21] Mirzaei S, Khaksary Mahabady M, Zabolian A, Abbaspour A, Fallahzadeh P, Noori M (2021). Small interfering RNA (siRNA) to target genes and molecular pathways in glioblastoma therapy: current status with an emphasis on delivery systems. Life Sci.

[22] Jain S, Pathak K, Vaidya A (2018). Molecular therapy using siRNA: recent trends and advances of multi target inhibition of cancer growth. Int J Biol Macromol.

[23] Mirzaei S, Gholami MH, Hashemi F, Zabolian A, Hushmandi K, Rahmanian V (2021). Employing siRNA tool and its delivery platforms in suppressing cisplatin resistance: approaching to a new era of cancer chemotherapy. Life Sci.

[24] Ji X, Lu Y, Tian H, Meng X, Wei M, Cho WC (2019). Chemoresistance mechanisms of breast cancer and their countermeasures. Biomed Pharmacother.

[25] Chen HK, Chen YL, Wang CY, Chung WP, Fang JH, Lai MD (2023). ABCB1 regulates immune genes in breast cancer. Breast Cancer (Dove Med Press).

[26] Kumari N, Dwarakanath BS, Das A, Bhatt AN (2016). Role of interleukin-6 in cancer progression and therapeutic resistance. Tumour Biol.

[27] Eichten A, Su J, Adler AP, Zhang L, Ioffe E, Parveen AA (2016). Resistance to anti-VEGF therapy mediated by autocrine IL6/STAT3 signaling and overcome by IL6 blockade. Cancer Res.

[28] Bocci G, Kerbel RS (2016). Pharmacokinetics of metronomic chemotherapy: a neglected but crucial aspect. Nat Rev Clin Oncol.

[29] Rugo HS, Bardia A, Tolaney SM, Arteaga C, Cortes J, Sohn J (2020). TROPiCS-02: a phase III study investigating sacituzumab govitecan in the treatment of HR + /HER2- metastatic breast cancer. Future Oncol.

[30] Capasso A (2012). Vinorelbine in cancer therapy. Curr Drug Targets.

[31] Krajnak S, Trier JP, Heinzmann PF, Anic K, Heimes AS, Loewe A (2023). Anti-tumor effects of low-dose metronomic vinorelbine in combination with alpelisib in breast cancer cells. EXCLI J.

[32] Ghosh S, Javia A, Shetty S, Bardoliwala D, Maiti K, Banerjee S (2021). Triple negative breast cancer and non-small cell lung cancer: clinical challenges and nano-formulation approaches. J Control Release.

[33] Meng H, Mai WX, Zhang H, Xue M, Xia T, Lin S (2013). Codelivery of an optimal drug/siRNA combination using mesoporous silica nanoparticles to overcome drug resistance in breast cancer in vitro and in vivo. ACS Nano.

